# Metastatic Cutaneous Melanoma of the Gallbladder

**DOI:** 10.1155/2017/8532379

**Published:** 2017-01-30

**Authors:** Dhruvan Patel, Shazia Sohrawardy, Yub Raj Sedhai, Soney Basnyat, Anisha Daxini, Aparna Basu, Vivek R. Mehta, Aasim Mohammed, Steven Lichtenstein

**Affiliations:** ^1^Internal Medicine, Mercy Catholic Medical Center, Darby, PA, USA; ^2^Philadelphia College of Osteopathic Medicine, Philadelphia, PA, USA; ^3^Nepal Medical College Teaching Hospital, Kathmandu, Nepal; ^4^JJM Medical College, Davangere, India; ^5^Hematology & Oncology, Henry Ford Medical Center, Detroit, MI, USA; ^6^Rheumatology, Albany Medical Center, Albany, NY, USA; ^7^Division of Gastroenterology & Hepatology, Mercy Health System, Philadelphia, PA, USA

## Abstract

Metastatic melanoma is an aggressive disease that can spread to many organs of the body. In rare cases, it can spread to the gallbladder causing secondary lesions, yet presenting with little to no symptoms. Therefore, most cases of metastatic melanoma lesions to the gallbladder go undiagnosed. Here, we present the case of a 41-year-old male with a four-month history of melanoma of the face, with a postresection status, who presented with right upper quadrant abdominal pain. Doppler ultrasound and computed tomography confirmed the presence of a mass on the gallbladder. Laparoscopic excision along with liver wedge resection was performed. Pathology staining revealed the presence of a malignant metastatic melanoma lesion of the gallbladder.

## 1. Introduction

Cutaneous melanoma is a very aggressive disease, arising from the proliferation of melanocytes, a type of dendritic cell found in the epidermis, uvea, meninges, intestinal tract, upper respiratory tract, and along regional lymph nodes [1–3]. While it comprises less than 5% of all skin cancer cases, it has an extremely high morbidity and mortality due to its high tendency to metastasize throughout the body [1–4]. The most common sites of distant metastases are the lungs, brain, liver, and GI tract [1–3]. Only 2–4% of patients affected by cutaneous melanoma are diagnosed with gastrointestinal metastases and the most common sites are the small bowel, colon, and stomach [1, 2]. Metastatic involvement of the gallbladder is extremely rare and, when present, is often part of a widespread complex of metastases with a very poor prognosis [1, 4, 5]. Interestingly, autopsy reports suggest gallbladder involvement in 4–20% of all cutaneous melanoma metastasis patients; however, they are associated with minimal to no symptoms, making diagnosis difficult during its lifetime [5–7].

Here, we report a rare case of melanoma metastasis to the gallbladder as the first site of recurrence following cutaneous melanoma treatment. The patient presented with right upper quadrant abdominal pain and was successfully treated with cholecystectomy and liver wedge resection surgery.

## 2. Case Report

Written consent for publication of this case was obtained from the patient. We present a 41-year-old male with a medical history of cutaneous melanoma of the face who presented to the hospital with abdominal pain. Three months prior to this admission, the patient was diagnosed with an ulcerated nodular malignant melanoma of his right temple, Breslow thickness 12 mm, invasive to Clark anatomic level IV. The patient underwent wide local excision of the lesion, with a negative sentinel node biopsy (Stage IIC, T4N0M0). Following excision of his facial lesion, he was started on interferon therapy 5 days a week. A one-month follow-up included a normal physical exam, normal complete blood count, and normal complete metabolic panel. Positron emission tomography (PET-CT) was also obtained at the same time and was negative for any metastatic lesions.

**Figure 1:**
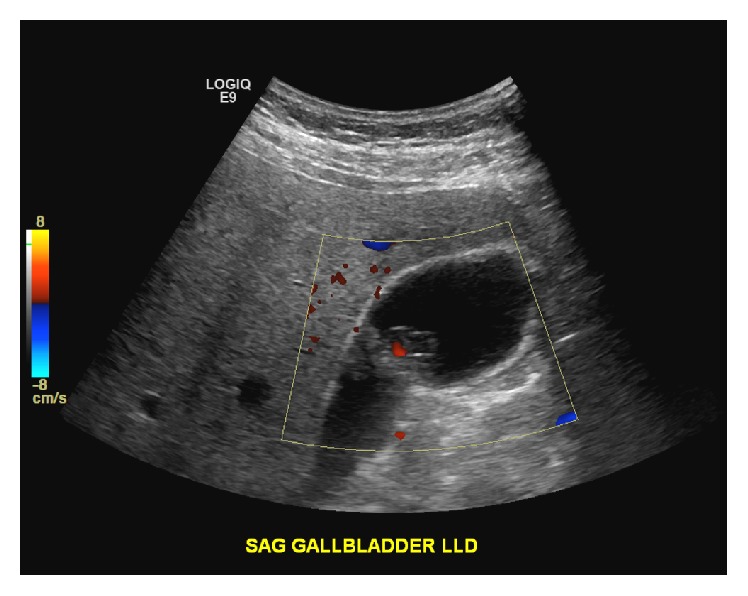
Ultrasound Doppler showing gallbladder mass with high blood flow.

**Figure 2:**
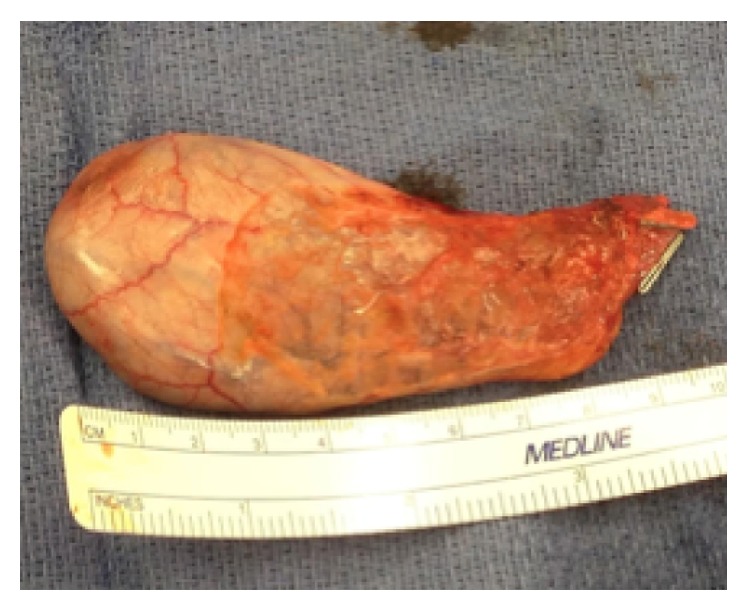
Surface of resected gallbladder.

**Figure 3:**
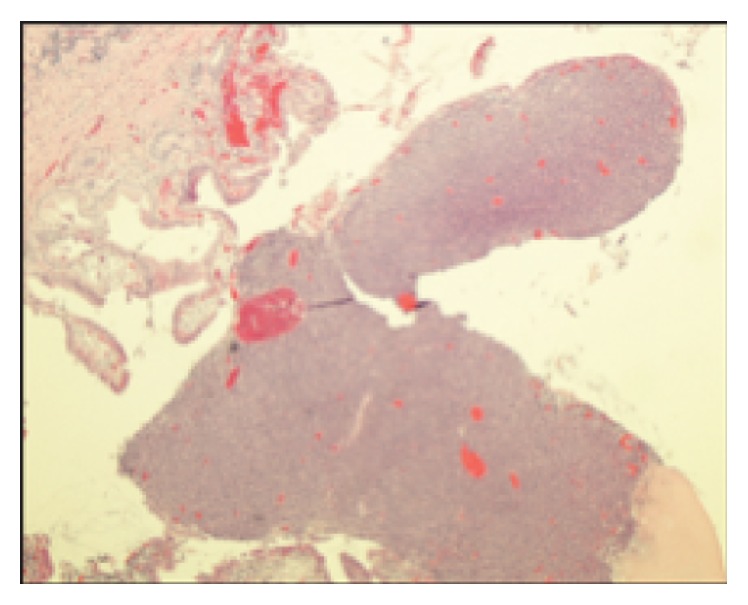
Pathology of gallbladder mass showing metastatic melanoma.

Three months after follow-up, the patient presented to the hospital with a 3-week history of right upper quadrant (RUQ) abdominal pain, nausea, alternating bowel movements, and a 15-pound weight loss. Physical exam revealed RUQ tenderness but was otherwise unremarkable. Laboratory testing showed his bilirubin, AST, and ALT to be 0.3 mg/dL, 62 U/L, and 96 U/L, respectively. An abdominal computed tomography showed a gallbladder lesion. Doppler ultrasound confirmed a 1.1 × 1 cm gallbladder lesion with high blood flow (Figure 1). Following this, an ultrasound-guided gallbladder biopsy was attempted but was unsuccessful because of an inability to locate the lesion precisely. Due to the high suspicion of metastasis, the patient underwent a laparoscopic cholecystectomy along with a hepatic wedge resection (Figure 2). This resection revealed no external evidence of a metastatic gallbladder lesion, both intraoperatively or on back table inspection. A frozen section of the gallbladder was also found to be negative for melanoma metastasis. There was no evidence of pigmentation within the abdomen. Despite all of this, pathology confirmed the suspicion of metastatic melanoma of the gallbladder through a positive immunohistochemistry stain, showing a BRAF V600K mutation (Figure 3). Our operative report justifies the presence of an intraluminal gallbladder mass, which can be easily missed on external appearance or on frozen section.

After surgery, the patient did not require adjuvant therapy, such as high-dose interferon or dabrafenib/trametinib, for his localized metastatic lesion, in accordance with the most recent guidelines for malignant melanoma [1, 5]. A follow-up PET-CT was obtained 1 month after surgery and showed no evidence of the disease recurrence. At the most recent follow-up, clinic evaluation 6 months after surgery, the patient was well without any evidence of recurrence.

## 3. Discussion

Melanocytes are a dendritic-type cell, which provides melanin to keratinocytes and can be found in most organ viscera, depending on neural crest migration during embryogenesis [1, 8]. Melanoma is a malignant tumor of melanocytes and represents <5% of all skin cancers [5, 9]. However, it has a high potential for widespread metastatic disease and thus is associated with high mortality. The incidence of melanoma is growing faster than any other potentially preventable cancer in the United States with an approximately 1.9 percent growth annually between 2000 and 2009 [10]. Globally, 132,000 new cases of melanoma will arise this year with 48,000 deaths per year [2].

Melanoma usually follows a benign course of early radial growth. The potential for metastasis greatly increases once the cutaneous lesion begins to spread vertically, penetrating blood and lymph vessels [1, 11, 12]. Tumor cells can spread to the local lymph nodes draining the region of the primary lesion. It can also travel hematogenously to distant sites, such as the soft tissues (50–75%), lung (70–87%), liver (54–77%), and brain [9, 11, 13]. Many authors have also postulated dissemination of tumor cells via bile, contributing to liver and GI tract invasion. Only 2–4% of patients affected by cutaneous melanoma are diagnosed with gastrointestinal metastases [8]. The most common sites for GI invasion are the small bowel (35–65%), colon (5–9%), and stomach (5–7%) [1, 2].

Cutaneous metastatic melanoma to the gallbladder is rarely found during a patient's life [9, 11, 14]. When found, it usually presents as part of a widespread metastatic disease. It is important to note that though travel to the gallbladder is rare, gallbladder melanoma accounts for 50–67% of all gallbladder tumors [11, 12, 15]. The prognosis is extremely poor for metastatic gallbladder melanoma with an average survival of 8.4 months and a 5-year survival of only 15% [9, 15, 16]. However, the prognosis of metastatic gallbladder melanoma drastically increases when the gallbladder lesion is a single focus of metastasis with no other lesions and is surgically removed [9, 13, 16]. Gallbladder metastases are relatively asymptomatic, which is why most cases are discovered during autopsy: Das Gupta et al. conducted an autopsy study on 125 patients and found that 15% (19 patients) presented with metastatic lesions to the gallbladder [6, 9, 11, 14]. In rare cases, melanoma metastasis to the gallbladder will present with symptoms such as abdominal pain, as was seen in our patient, or as an acute cholecystitis [11, 15]. Patient clinical presentation may include jaundice from obstruction of the bile duct by the tumor mass, hemobilia, biliary fistulas, vomiting, nausea, and weight loss [5, 7, 14].

The rarity of the metastasis combined with its asymptomatic nature means the widespread disease has usually occurred by the time of diagnosis [5, 9, 13]. Addressing this involves maintaining a high level of suspicion in patients who present with biliary symptoms and have a known history of cutaneous melanoma [2, 8]. Doppler ultrasonography remains to be the method of choice for determining the presence of a gallbladder malignancy by detecting high blood flow regions [7, 13, 15]. In cases of gallbladder melanoma, ultrasonography will show single or multiple infiltrative lesions (at least 1 cm in diameter) attached to the inner mucosal wall [3]. Computed tomography can also be utilized and has 60–70% sensitivity in detecting metastases [2]. Positron emission tomography can be utilized secondarily in detecting the extent of spread [1, 2]. Finally, biopsy of the gallbladder lesion and immunohistochemical staining are used to confirm the diagnosis [2].

Surgery, as a treatment modality for metastatic melanoma to the gallbladder, remains questionable since most cases of gallbladder melanoma are a part of widespread disease [1]. Metastasectomy remains to be the mainstay approach, for its palliative and symptom-reducing effects and for cases of a single localized lesion to the gall bladder like in our patient [7, 9, 15]. Surgery also functions to prevent further dissemination from the gallbladder via bile [1]. Dong et al. found that the 1-year survival in surgically resected patients can be up to 100%, but without surgery it is documented at as low as 0% [9]. A liver wedge resection, targeting nearby regions of the liver pedicle, should also be performed in cases of probable contiguous spread of neoplastic cells [8].

Management of the patient can also involve chemoimmunotherapy, especially in the case of a nonlocalized lesion [1, 2]. Its utility as adjuvant therapy remains unclear [1–3]. Chemoimmunotherapy has garnered recent interest as an effective treatment modality. Among them is the use of high-bolus interleukin-2 (IL-2) which has been found to induce remission in 15% of patients; however, its use is limited due to toxicity [2, 8]. BRAF inhibitors along with immune therapy have been utilized in generating an immune response to the tumors [2, 8]. The use of MEK inhibitors concurrently with BRAF inhibitors has been shown to lower adverse reactions, prolong disease-free survival, and delay the resistance that has been seen in BRAF inhibitor use alone [1].

Melanoma undergoes extensive and rapid growth and causes widespread damage with very high morbidity and mortality. We have presented this case to address the need for a prompt workup with a potential diagnosis of metastasis, especially in patients with a known history of cutaneous melanoma. The key to prolonging survival is to consider metastases early and begin aggressive treatment. Most cases of gallbladder metastases present with no symptoms. Workup should begin with imaging, along with immunohistochemical staining for confirmation. Adequate treatment may be achieved with laparoscopic cholecystectomies and/or chemoimmunotherapy.
